# The Gene Regulatory Roles of Herbal Extracts on the Growth, Immune System, and Reproduction of Fish

**DOI:** 10.3390/ani11082167

**Published:** 2021-07-22

**Authors:** Ehsan Ahmadifar, Hamideh Pourmohammadi Fallah, Morteza Yousefi, Mahmoud A. O. Dawood, Seyed Hossein Hoseinifar, Hossein Adineh, Sevdan Yilmaz, Marina Paolucci, Hien Van Doan

**Affiliations:** 1 Department of Fisheries, Faculty of Natural Resources, University of Zabol, Zabol 98613-35856, Iran; Ehsan.Ahmadifar@uoz.ac.ir; 2 Department of Medicine, University of British Columbia, Vancouver, BC V1Y 1T3, Canada; hamideh.pourmohammad@ucalgary.ca; 3 Department of Veterinary Medicine, Peoples’ Friendship University of Russia (RUDN University), 6 Miklukho-Maklaya St, 117198 Moscow, Russia; myousefi81@gmail.com; 4 Department of Animal Production, Faculty of Agriculture, Kafrelsheikh University, Kafrelsheikh 33516, Egypt; mahmouddawood55@gmail.com; 5 Department of Fisheries, Faculty of Fisheries and Environmental Sciences, Gorgan University of Agricultural Sciences and Natural Resources, Gorgan 4918943464, Iran; hossein.hoseinifar@gmail.com; 6 Department of Fisheries, Faculty of Ariculture and Natural Resources, Gonbad Kavous University, Gonbad Kavous, Golestan 4971799151, Iran; Adineh.h@gonbad.ac.ir; 7 Department of Aquaculture, Faculty of Marine Sciences and Technology, Canakkale Onsekiz Mart University, Canakkale 17100, Turkey; sevdanyilmaz@comu.edu.tr; 8 Department of Science and Technologies, University of Sannio, 82100 Benevento, Italy; paolucci@unisannio.it; 9 Department of Animal and Aquatic Sciences, Faculty of Agriculture, Chiang Mai University, Chiang Mai 50200, Thailand

**Keywords:** herbal extracts, gene regulations, immunity, growth, aquaculture

## Abstract

**Simple Summary:**

The maintenance of animal health and preventing disease is an important point for sustainable aquaculture. In this sense, medicinal herbs can help farmers to achieve this goal. Although many review articles have discussed the effects of these additives on growth performance, immune and antioxidant responses, as well as resistance to diseases in fish, limited knowledge is available on how herbs affect these parameters at the molecular level. Given that studies at the molecular level can provide an accurate picture of the mode of action of feed additives, in this paper, the results of studies focusing on the effect of herbs on the expression of different genes in fish are discussed.

**Abstract:**

The crucial need for safe and healthy aquatic animals obligates researchers in aquaculture to investigate alternative and beneficial additives. Medicinal herbals and their extracts are compromised with diverse effects on the performances of aquatic animals. These compounds can affect growth performance and stimulate the immune system when used in fish diet. In addition, the use of medicinal herbs and their extracts can reduce oxidative stress induced by several stressors during fish culture. Correspondingly, aquatic animals could gain increased resistance against infectious pathogens and environmental stressors. Nevertheless, the exact mode of action where these additives can affect aquatic animals’ performances is still not well documented. Understanding the mechanistic role of herbal supplements and their derivatives is a vital tool to develop further the strategies and application of these additives for feasible and sustainable aquaculture. Gene-related studies have clarified the detailed information on the herbal supplements’ mode of action when administered orally in aquafeed. Several review articles have presented the potential roles of medicinal herbs on the performances of aquatic animals. However, this review article discusses the outputs of studies conducted on aquatic animals fed dietary, medicinal herbs, focusing on the gene expression related to growth and immune performances. Furthermore, a particular focus is directed to the expected influence of herbal supplements on the reproduction of aquatic animals.

## 1. Introduction

The provision of food for human beings is one of the main challenges facing humanity, which has attracted the attention of different countries to increase the number of aquaculture products in their food basket. Besides, high-quality proteins derived from aquaculture products have made the aquaculture industry, with an annual growth of 8%, the highest activity in the food industry [1], and in the last decade, global aquaculture has increased by 163%, reaching 114.5 million tons in 2018 [1]. Fish, both salt and fresh water are healthy and high-quality foods because they contain valuable nutrients such as vitamins, minerals, high protein content, essential amino acids, and fatty acids. During recent years, growth performance and artificial reproduction have been considered as two primary concerns in aquaculture. Increasing stock densities in limited areas to achieve more production in line with the increasing demand has resulted in the increase of organic load, which impairs water quality in the environment, and the imbalance of water parameters such as dissolved oxygen and pH important for fish health. This has triggered the fish to get stressed and contract diseases more easily. Chemotherapeutics and antibiotics have been utilized for many years to prevent stress and combat diseases in aquatic animals [2]. However, their excessive and improper use suppressed the immune system of hosts and caused resistance against pathogenic microorganisms.

The aquaculture industry has applied different strategies to eliminate or reduce mortality from various diseases. One of the strategies is to use herbal extracts to improve the immune state. Among such strategies, these is the enhancement of the immune status of the cultured aquatic organism with immunostimulants. In aquaculture, herbal extracts, phytochemicals, and plant secondary metabolites are important feed additives that activate the specific or nonspecific immune system. In the literature, there have been many studies on the effects of Phyto-additives on cellular, humoral, and adaptive immune responses in fish [2,3,4,5]. In recent studies, there has been a trend toward investigating the effects of Phyto-additives on cellular or textural gene expression responses to understand the mechanisms of Phyto-additives in fish.

In fish, growth hormone (*GH*) and insulin-like growth factor, I (*IGF-I*) play a central role in regulating growth. Associated with reproduction, the presence of various genes including luteinizing hormone β (*lhβ*), follicle-stimulating hormone β (*fshβ*) [6], estrogen receptors (*erα*, *erβ1*, and *erβ2*), androgen receptors (*arα* and *arβ*) [7], vitellogenins (*Vtgs*) [8], aromatase genes [9] has been confirmed. Although the number of studies is limited, Phyto-additives have been reported to affect expressions of growth genes such as *GH* and *IGF* and reproductive genes such as *fshβ*, *lhβ*, *cyp19a*, and *vtg* in fish.

Another effect of Phyto-additives is increasing the secretion of digestive enzymes. Thus, the transfer of nutrients from food to the body increases, and this affects fish development positively. The immunomodulatory, disease resistance-enhancing, digestive enzymes activating, growth-enhancing, and reproduction stimulating characteristics of Phyto-additives is linked to the active compounds they contain [10]. Among these compounds, alkaloids, essential (volatile) oils, flavonoids, glycosides, lectins, phenolics, quinones, polyphenolics, polypeptides, polysaccharides, saponins, steroids, tannins, and terpenoids are the most important. Innate and adaptive immune systems interact through cytokines which regulate host immune responses against disease [11]. Phyto-additives have shown an immunostimulatory effect by up-regulating the expressions of the proinflammatory cytokines *IL-1β,* *TNF-α, IL-6,* and *IFNγ* and downgrading the expressions of anti-inflammatory cytokines including *IL-1*, *IL-4*, *IL-10*, *IL-11*, and *IL-13*.

The number of aquatic species for artificial reproduction and farming is on the rise owing to the development of commercial aquaculture. A prerequisite for the artificial reproduction and sustainable production of fish is the control of the reproductive process of fish in captivity and the production of high-quality sperms and eggs. Different studies showed the effect of Phyto-additives on reproductive processes.

In this review, studies related to the effects of Phyto-additives on the expressions of the genes associated with immunity, digestion, growth, and reproduction were reviewed.

## 2. The Effect of Phytochemicals and Their Derivatives on Growth-Related Genes

Growth is a polygenic and environmentally controlled trait that is defined as a somatic function that reflects the balance between feed composition and quality, consumption, utilization, and the physiological functions of an organism [12]. Many factors, including genetics, nutrition, and the environment can affect the growth rate of an organism. Feed additives are the substances that are used in the diet of animals in small amounts to improve the effectiveness and absorption of nutrients in the intestine [13]. In this way, these materials can increase the growth efficiency as well as the health of farmed organisms [11]. In recent years, the use of phytochemicals as natural growth promoters has been increased in animal husbandry and aquaculture [10,14,15,16]. The application of phytochemicals and their derivatives as immune stimulators and growth promotors in aquatic animals has been well-reviewed [10,14,17,18], however, there is a lack of information on the mode of action of these components in the pathways, affecting growth-related genes in aquatic organisms. Thus, we have attempted to provide an overview of the effect of phytochemicals and their metabolic components on growth-related genes in aquatic organisms (Table 1).

Growth hormone (*GH*) and insulin-like growth factor-I (*IGF-I*) are considered the main genes influencing the growth that form the core of the hypothalamic-pituitary–somatotropic (HPS) axis. These genes are influenced by several factors such as the environment, genetics, and nutrition of an organism [12]. Growth hormone has direct and indirect metabolic effects. In direct mode, *GH* in a series of steps enhances protein syntheses, including synthesis of RNA and amino acid uptake. Indirectly, after being secreted from the pituitary gland, *GH* circulates through the blood to the liver, where it stimulates the synthesis and secretion of *IGFs* such as *IGF-1* and *IGF-2* [20,23]. In addition, *GH* may boost the local synthesis of *IGF-1* that exerts paracrine or autocrine effects [23]. Subsequently, *IGF-1* by acting on target cells, causes them to proliferate and differentiate and ultimately stimulates the growth of the body [24]. The previous studies on the Nile tilapia showed that there is a positive correlation between IGF-1 protein and mRNA levels in liver and body weight gain and SGR of this fish species [25,26]. In addition to these hormones, other factors play a role in fish growth. For example, fish intestine plays the most important role in the digestion and absorption function of the gastrointestinal tract and thus show a substantial effect on fish nutrition and growth [20]. The profile and activity of an animal’s digestive enzymes largely characterize its capacity to absorb nutrients from a particular food source [27]. It has been demonstrated that there is a significant correlation between increased production of digestive enzymes (mainly included a-amylase, protease, and lipase) and digestive capacity [20] and growth [28]. Moreover, the expression of several genes such as mucin-like protein (*muc*), oligo-peptide transporter I (*pept1*), and lipoprotein lipase (*lpl*) enhance digestion, absorption, and transport of nutrients in the intestine [21]. These functions are essential for the efficient utilization of dietary components and ultimately lead to enhanced growth of the animal. For example, *muc* gene plays a role in secreting the mucus needed for the efficient transport of nutrients from the intestine into the blood [29,30]. Pept1 is known as a nutrient transporter that actively transports di- and tripeptides from enterocyte cells into the bloodstream, using mucus as a mediator [31]. Diets containing di- and tripeptides have been shown to increase fish growth more efficiently than individual amino acids [31,32]. Lpl is an enzyme that breaks down plasma lipids and releases fatty acids, which in turn are transported to tissues through the bloodstream to produce energy [33].

Phytochemicals and their derivatives can exert prebiotic-like effects in fish by acting as selective growth factors and fermentation substrates for advantageous bacteria, while preventing the development of pathogenic bacteria in the gastrointestinal tract [2,34]. Polyphenols have also been shown to stimulate the activity of digestive enzymes and increase the synthesis of DNA, RNA, and proteins, as well as GH and IGF-1 production and function. Moreover, they stimulate the expression of genes involved in the appetite and food intake (*ghrelin*; *GHRL*), absorption and transport of nutrients (*muc* and *pept1*), and assimilation of lipids (*lpl* and *alp*), as well as exhibit other metabolic functions by enhancing intestinal microbiota, all of which in turn increase growth [16,19,20,21,35]. In addition, phytochemicals have been shown to stimulate the animal’s immune system (both specific and non-specific) via enhancing the immune cell functions, increasing cytokine and antibody productions, and improving immune-related gene expression. These beneficial functions of phytochemicals subsequently lead to better health, disease resistance, and thus faster growth of fish [16,36].

The following are some of the studies that have examined these beneficial effects of the phytochemicals and their derivatives on the expression of growth-related genes. Tannins are in the category of polyphenolic compounds found in many terrestrial plants and aquatic macrophytes. They are available as hydrolysable tannins, condensed tannins, and chlorotannins that differ in chemical structure [37,38]. A study on beluga sturgeon (*Huso huso*) showed that 0.05 or 0.1% dietary tannin extracted from chestnut wood (Silvafeed^®^ATX), after a six-week feeding trial, enhanced the growth performance, some immune parameters, and expression of *gh* and *igf-i* genes in this fish species. The authors suggested that tannin might improve fish growth by regulating GH/IGF-1 axis [19].

Curcumin is a polyphenolic compound found naturally in the turmeric plant rhizome (*Curcuma longa*). It contains biologically active compounds such as alkaloids, triterpenoids, and reducing sugars that have immune-modulating properties and act as prebiotics [39]. Curcumin can strengthen the balance of positive and negative gut flora and increase intestinal digestion and absorption, thereby stimulating general health and increasing fish growth [39]. Midhum et al. [20] examined the modulating effects of the dietary curcumin on digestive enzymes, and the expression of *gh*, *igf-1*, and *igf-2* genes in tilapia (*Oreochromis mossambicus*). They found that 0.5 and 1% curcumin inclusion significantly increased *gh* in the brain, and *igf-1* and *igf-2* gene expression in muscles. These genes, along with *myostatin*, are involved in fish myogenesis and have been shown to act as extrinsic regulators and control fish muscle growth [39].

D-limonene [1-methyl-1-4-(1-methylethyl) cyclohexane], is a natural phytogenic compound that is found in cherries, grapes, and citruses such as lemon and orange [40]. It has hypoglycemic activity, antioxidant properties as well as chemo-preventative activity against cancers and many other diseases [41,42]. Sharma and Bansal [43], reported that D-limonene successfully modulates streptozotocin-induced insulin secretion and lowers lipid peroxidation in diabetic rats. Dietary limonene at inclusion levels of 400 and 600 ppm was shown to increase weight gain and up-regulate the expression of *igf-1*, *muc*, *pept1*, *lpl* and *alp* in the Nile tilapia (*O. niloticus*) [21]. Such results suggest that limonene by activating these genes can enhance the absorption and transport of nutrients and assimilation of lipids, which ultimately increase fish growth.

Apple cider vinegar mainly contains organic acids, minerals, some vitamins, and flavonoids. Ahmadifar et al. [22] found that dietary apple cider vinegar at 3 and 4.5% inclusion levels significantly up-regulated the expression of ghrelin (*GHRL*) in zebrafish (*Danio rerio*). *GHRL* is known as an appetite stimulant gene that is involved in *GH* secretion, food intake, and energy homeostasis.

It seems that the mode of action of phytochemicals and their derivatives involves up-regulating the expression of growth-related genes, which activate a series of functions and eventually improve fish growth.

## 3. The Effect of Herbal Extracts and Plant Components on Immune-Related Genes in Fish Species

Non-specific immunity is characterized by recognition of non-self molecules [44] to identify pathogen-associated molecular patterns (PAMPs) and kill the pathogens via cellular immunity [45]. Resistance to pathogens is an important part of the innate immune response. Since many pathogens, including bacteria, viruses, and parasites, can infect fish species in the aquaculture sector, the use of effective and safe treatments is of paramount interest for the aquaculture industry [46]. Antimicrobial agents have been used in aquaculture for decades to prevent microbial diseases and can be classified as either synthetic or natural. The use of synthetic antimicrobial agents or antibiotics has resulted in inherited mutations in pathogenic microorganisms and resistance to these agents. Several studies have described the diversity and prevalence of antibiotic resistance in fish farms [47]. The use of high-dose antibiotics is not only ineffective for disease control in fish farms, but also endangers human health and environmental sustainability by killing environmental bacteria when administered on a long-term basis [48]. Alternatively, natural components from plants are generally recognized as safe substances (GRAS) due to their few side effects and advantageous performance; therefore, they can be used as therapeutic agents to treat infections in aquaculture [49].

Herbal bioactive components can act as immunostimulants and influence several immune-related pathways (Table 2). An immunostimulant is a component or action that elevates immune responses, especially innate immunity [50]. Herbal bioactive components can have anti-bacterial, anti-viral, and anti-fungal functions and increase resistance against infectious microorganisms [36]. Secondary metabolites (SM) in the plants, including anti-microbial polypeptides, polysaccharides, phenolic compounds, essential oils, and saponins, have been found to have high potency to treat infections and low toxicity [51]. These compounds can act as modulators of active sites, receptors, and catalytic sites of enzymes and active proteins to treat diseases [52].

Several herbal compounds have been shown to modulate immune responses and treat infections. Plants from the Lamiaceae family (mints) have been studied in several recent experiments [60,66,67,68,73,74]. One study by Abdellatif et al. reported a significant effect of combined sage (*Salvia officinalis*) and *Spirulina platensis* (*Arthrospira platensis*) on levels of immune markers, including lysozyme, nitric oxide, and immunoglobulin M (IgM), as well as increased resistance against *Pseudomonas aeruginosa* in Nile tilapia [68]. Another study revealed a significant effect of 10 g/kg rosemary (*Salvia rosmarinus*) leaf on serum catalase and lysozyme activities, the alternative complement pathway, and resistance against *Aeromonas hydrophila* in the Nile tilapia [73]. In addition to the aforementioned findings, bioactive components found in the Lamiaceae family can induce immune responses under toxic effects of water pollutants, such as chlorpyrifos, that activate inflammatory pathways and suppress effective immune responses in fish and other marine species. Dawood et al. showed a significant reduction in gene expression of pro-inflammatory cytokines (interleukin [*IL*]-*8* and *IL-1β*) in gills, liver, and intestine of teleost species and increased activity of antioxidant enzymes (catalase, superoxide dismutase) after supplementation with menthol oil [60]. Because the essential oils found in the Lamiaceae family have been studied as a potential component to elevate immune responses and prevent infections, several studies have been conducted to understand the molecular mechanisms and clinical efficacy. For example, Zargar et al. [66] found that supplementation with *Thymus vulgaris* essential oils up-regulated transcription of immune-related genes. Although the efficacy of the intervention was different at various concentrations, the dose of 1 mL/kg diet increased gene expression of complement component 3 (C3) and the cluster of differentiation 4 (CD4). A higher dose of 2 mL/kg enhanced lysozyme transcription levels. These findings were not consistent at the final time point: both concentrations increased gene expression of C3 and CD4, but the 2 mL/kg concentration reduced lysozyme and *IL-1β* gene expressions [66]. In addition, Serradell et al. conducted an experiment investigating the effects of concurrent use of essential oils from the Lamiaceae family (200 ppm) and plant galactomannan oligosaccharides (500 ppm) on stress and immune gene expressions in European sea bass [55]. The findings revealed a decrease in *cyp11b*, *hif-1α*, *casp-3*, and *il-1β* gene expression and increases in steroidogenic acute regulatory protein (StAR) gene expression and serum lysozyme activity [55]. The essential oils can have a prophylactic role; likewise, leaf extracts demonstrate a similar effect. In a study by Salomón et al. [56] a combined leaf extract from *Salvia officinalis* and *Lippia citriodora* elevated the expression of genes involved in humoral immunity and inflammation, as well as leucocyte cell surface markers and antioxidant enzymes in gilthead seabream. This treatment did not exhibit any significant effect in non-induced pancreatic cells, and the significant findings were recorded after challenge with lipopolysaccharide. Another component extracted from essential oils is citral, which has been shown to exert anti-inflammatory and antibacterial effects due to its monoterpenoid structure [75,76]. Moreover, it can be used as a natural anesthetic agent in animal breeding [77]. Mori et al. reported a beneficial role of citral in inducing certain immune-related indicators, including lipoperoxidation and antioxidant enzyme levels; however, it did not affect lysozyme activity [78].

By the same token, polyphenols have been described as anti-inflammatory [79], anti-microbial [80], and anti-oxidant [81] bioactive compounds. An example in this regard is trans-cinnamic acid, which has an immunostimulant role via activation of pro-inflammatory cytokine gene expression, including *IL-1β*, *IL-8*, transforming growth factor-beta (*TGF-β*), tumor necrosis factor-alpha (*TNF-α*), IgM, and *IgT* [69]. The findings on head kidney specimens of rainbow trout were consistent with the previous studies in other fish species; adding 250 or 500 mg/kg feed resulted in up-regulation of gene expression levels of head kidney pro-inflammatory cytokines after 60 days [69].

On this subject, a study by Wang et al. [53] suggested a beneficial role of *Glycyrrhiza uralensis*, a species from the Fabaceae plant family, in immune response and resistance against *Flavobacterium columnare* in yellow catfish. Supplementation with the extracts increased gene expression of toll-like receptor (*TLR*) 2, *TLR3*, *TLR5*, *TLR9*, *Myd88*, and *p65NFκB*, and subsequently increased *IL-1β* and *IL-8* gene expressions. The significance level was demonstrated only at the post-infection time point in head kidney specimens, not at the other time points. This indicates that the timing of supplementation matters to a significant extent for the supplement to act as an effective medication [53,82]. These traditional herbal medicines have been used to treat several inflammatory diseases in clinical investigations, but fewer studies have aimed to investigate their beneficial roles in marine species. A combination of three herbal medicines was investigated in a study by Cai et al. that reported an up-regulation in differentially expressed genes (DEGs) of pathways associated with infections and immunity in tiger grouper species: *Spatholobus suberectus*, *Phellodendron amurense*, and *Eclipta prostrata* showed an immunoregulatory role via modulation of *TLR5*, *IL-8*, and mitogen-activated protein kinase (*MAPK*) pathways [82].

Chaihu (*Radix bupleuri*), Bangkal (*Nauclea subdita*), Buton (*Eleutherine bulbosa*), Stinging nettle (*Urtica dioica*), and contain phytochemicals such as flavonoids, triterpenoids, sterols, essential oils, lignans, tannins, alkaloids, and phenolics. These phytochemicals can improve immune dysregulation consequences of hyperlipidemia, increase levels of lymphocytes, phagocytosis, and defense against *A. hydrophila* infection, and act as antioxidant, anti-inflammatory, and anti-viral components. In addition to these, the herbs also up-regulated gene expression levels of *MHC2*, *IKKα*, *TGF-β1*, *TNF-α*, *IL-1β*, *IL-6*, and *IL-8* (93, 64, 94, 95, 96, 97, 98, 99, 101, 102).

In addition to the aforementioned herbs, another health-promoting plant is fenugreek (*Trigonella foenum graecum*), which has been shown to have anti-inflammatory, anthelmintic, and immunostimulant properties in several fish species including common carp [83], striped catfish [84], gilthead seabream [85], and Nile tilapia [86]. Fenugreek is a rich source of polyphenols, vitamins, polypeptides, and polysaccharides. It has been reported to modify enzymatic activities and immune response pathways. A study by Moustafa et al. showed an up-regulation in gene expression of *IL-1β* and *TNF-α*, reduced levels of aspartate aminotransferase (AST), and alanine aminotransferase (ALT), and increased resistance against *A. hydrophila* after administration of 3% fenugreek seed powder in Nile tilapia [86,87].

Supplementation with a natural antibiotic called snogga wood (*Strychnos ligustrina Bl.*) at a dose of 10% per feed elevated phagocytosis and leukocyte count and showed activity against *Streptococcus agalactiae*; however, it did not alter hematocrit and leukocrit levels in Nile tilapia at any dose examined [88]. Pawpaw or papaya (*Carica papaya*) is a tropical plant from Central America and southern Mexico that contains unlike groups of bioactive agents, including saponins, flavonoids, carotenoids, and proteolytic enzymes. In vitro and in vivo research has demonstrated antibiotic, anti-inflammatory, and immunomodulatory effects of this tropical plant [89,90]. Fluted pumpkin (*Telfairia occidentalis*) is a rich dietary source of phytochemicals, especially tannins. This ancient medicinal herb has been shown to exert antimicrobial effects via growth inhibition of common microorganisms and an immunostimulant role [91]. A recent study has shown a beneficial role of combined pawpaw and pumpkin leaves in enhancing resistance to common bacterial infections in fish. Fakoya et al. conducted a study that revealed a strong antimicrobial effect of a combined extract of pawpaw and fluted pumpkin in doses of 425 μg/mL and 850 μg/mL against the growth of eight common fish pathogens [92].

*Jatropha* species have been used as traditional medicine for prophylaxis and treatment of various clinical disorders in tropical regions. *Jatropha vernicosa* is a recently registered species, and a recent screening study was conducted to investigate its antioxidant and immune-related features in longfin yellowtail fish [54]. This plant has been evaluated as a rich source of many phytochemicals including flavonoids, saponin, and coumarin [93]. The stem bark extract of *J. vernicosa* demonstrated antioxidant and immunostimulant effects via activation of respiratory burst, phagocytosis, nitric oxide synthesis, up-regulation of pro-inflammatory cytokines, and down-regulation of anti-inflammatory markers, resulted in control of vibriosis in spleen leukocytes [54].

Plant alkaloids, such as sanguinarine, (SG) exert toxic effects in high doses by inhibition of Na⁺/K⁺-ATPase in membranes of animal cells [94] and show a beneficial role in low doses [95]. *Macleaya cordata* (Willd) *R. Br.*, *Eomecon chionantha Hance*, and *Chelidonium majus* are the main sources of SG [96]. SG has a high cell permeability and can inhibit many pathways in animal cells [97]. Administration of 0.8 g/kg SG reduced the levels of gene expression of proinflammatory cytokines, including *TNF-α*, *IL-1β*, *TLR/-4*, *TLR-7*, and *TLR-8*. Interestingly, higher inclusion of cottonseed and rapeseed meal was negatively associated with immune-related gene expression in the intestinal mucosal barrier and survival after bacterial challenges [97].

Traditional herbal medicine from southeast Asia is the *Myrtaceae* family. An example is Aiton (*Rhodomyrtus tomentosa*), which has been reported to be an anti-inflammatory agent in the treatment of various diseases [98,99,100]. Adding Aiton extract in 10 μg/mL doses has been shown to reduce inflammatory mediators and enhance antioxidant status and anti-inflammatory markers in rainbow trout macrophages [72].

The *Moringaceae* family has been considered to treat several animal diseases as these plants are rich in various phytochemicals including carotenoids, flavonoids, and xanthins, as well as vitamins such as vitamins C and A [101]. Different parts of the Moringa plant have been used for pharmacologic objectives, and the leaf is a popular part to use in these medications. They can potentially exhibit miscellaneous functions as immunostimulants and bactericides [102]. A study of supplementation with various concentrations of Moringa leaf meal augmented phagocytosis, respiratory burst, and peroxidase levels in gilthead seabream [71]. The authors suggested that a 5% dose of Moringa leaf enhanced mucosal immune-related gene expression in the skin and intestine, including antioxidant enzymes, C3, and anti-inflammatory cytokines. Another study from these researchers concluded that a 5% dose can be useful to reduce the negative effects of hydrogen peroxide on mucosal immune markers, including phosphatase, peroxidase, protease, antiprotease, and lysozyme activity as well as IgM levels in seabream gill and intestine [57].

In addition to the abovementioned components, blue-green algae or spirulina can be a potential feed supplement for the health and welfare of marine species. Pectin, the main ingredient of the cell wall in spirulina, showed an immunomodulatory effect in zebrafish via up-regulation of pro-inflammatory cytokines, chemokines, lysozyme, and mucin as a medication against *E. piscicida* and *A. hydrophila* [67]. The combination of spirulina with selenium nanoparticles can trigger immune responses via activation of liver *SOD* and *TNF-α* gene expressions and suppression of *HSP70* in Nile tilapia [58].

## 4. The Effect of Herbal Extracts and Phytochemicals on Reproduction-Related Genes

Reproduction is a physiological procedure known to be regulated by a complex coordination of hormones at the hypothalamus, pituitary, and gonadal (HPG) levels. In vertebrates, hypothalamic gonadotropin-releasing hormone (GnRH) stimulates the synthesis and secretion of gonadotropins from pituitary cells [103]. Gonadotropins (luteinizing hormone β (*lhβ*) and follicle-stimulating hormone β (*fshβ*)), in turn, regulate gonadal function, that is, gametogenesis and hormone production. Recent studies performed in zebrafish demonstrated that GnRH isoforms locally produced in the gonads could also directly act on testis and ovary and regulate gametogenesis in vitro in an autocrine/paracrine manner [104]. In addition to GnRH-induced gonadal function, numerous environmental factors could also affect reproduction. Many studies as discussed below have provided evidence for linkage between herbal extracts and plant secondary metabolites (PSM) with physiological or pathophysiological conditions associated with reproduction. These herbal extracts and PSMs have been shown to alter fertility by changing the levels and activities of several hormones on the HPG axis. This alteration is known to be in part regulated by induced changes in the expression of reproductive-related genes. For instance, it has been demonstrated that phytochemicals could act on fish reproductive function by decreasing estradiol concentration through inhibition of aromatase enzyme (*cyp19a*) or reducing the bio-conversion of testosterone to estradiol [105,106]. Besides, it has been shown that phytochemicals could affect fish reproduction and prevent the synthesis of vitellogenin (*VTG*) by binding to the estrogen receptor instead of estradiol [107]. Changes in the transcript levels of ERs in the liver are closely related to the regulation of vitellogenin synthesis in most teleosts [65,108]. VTGs are synthesized and secreted by the liver during estrogen stimulation and then transported to the ovary through the blood, taken up by oocytes, and converted into phospholipid-rich yolk proteins [8]. In addition to the above-mentioned genes, alteration of the expression levels of estrogen receptors (ERs) such as *erα*, *erβ1*, and *erβ2* [108], and androgen receptors including *arα* and *arβ* [7], have been reported in fish. Other important genes having changes in the expressions of reproductive functions include aromatase (*cyp19a* and *cyp19b*). *cyp19a* plays a role in female gender determination and differentiation, while *cyp19b* shows high expression in both sexes in adult fish [9]. In addition, the presence of other genes associated with steroidogenesis such as *star*, *cyp11a1*, *cyp17a1*, *3β-hsd*, *11β-hsd2*, *17β-hsd3*, and *ftz*, androgen receptor (*ar*), sex-determining region Y-box 9a (*sox9a*), and double-sex and mab-3 related transcription factor 1 (*dmrt1*) have been reported in fish. Other than the referred reproduction-related genes affected by herbal extracts and PSMs in fish species, there are considerable numbers of genes affected in higher vertebrates. It is also acknowledged that the integrated regulation of reproduction can be altered through changes in apoptotic mechanisms, at different levels of the HPG axis. Here, we summarized the effects of a number of herbal extracts and PSMs on reproduction-related genes at different levels of the reproductive axis (Table 3) in different species of fish.

In addition to the aforementioned compounds, genistein, and daidzein, two natural Phyto-estrogens found in plants, affect reproductive processes depending on the dosage used, fish species, and age [118]. Schiller and colleagues exposed zebrafish embryos to genistein at 2.4 mg/L (EC10) for 48 h. They also exposed medaka embryos to genistein at 6 mg/L (EC10) and 10 mg/L (EC20) for 7 days [111]. Results showed that in both zebrafish and medaka *cyp19a1b* and *vtg1* gene expressions increased, while a decrease in the expression level of the *cyp19a1a* gene was only found in medaka. In a different study, genistein was injected intraperitoneally to *Dicentrarchus labrax* (immature; 59.4 g ± 0.7) fish at a dose of *5 mg/kg,* and after 24 h, scale and liver *vtg2* and *chgl* gene expressions increased. At the end of the 5th day, similar results were found only in the liver tissue [110]. In a very recent study using the *Cyprinus carpio* fish model it was found that ovary *cyp19a1a*, and liver *vtgb2*, and *erβ* gene expressions decreased after feeding female *Cyprinus carpio* fish with 0.01, 0.03, 0.06, and 0.09 g/kg genistein supplements for 60 days [109]. Moreover, other studies in zebrafish (embryos-larvae) showed that exposure of fish to genistein and daidzein at a concentration of 1.25, 2.5, 5, 10, and 20 mg/L for 96 h, increased expressions of *esrrb* and *cyp1a* [113]. Adult male and female zebrafish were also exposed to 10 mg/L genistein and daidzein concentrations for 10 days [112]. Results showed that in genistein exposure, HE1 gene expression increased in both ovary and testis, while only the ovary showed a decrease in *erβ*. Moreover, only testicular BRDT gene expression changed in the daidzein exposure [112]. Apart from the above-mentioned studies, another research performed in *Oncorhynchus mykiss* juveniles showed that injection of 5 μg/g body weights of genistein and daidzein along with 50 μg/g body weight genistein to fish, for 24 h, liver vtg, and *era1* gene expressions increases [114]. Equol, on the other hand, is a nonsteroidal estrogen, metabolized from daidzein. It has been shown that this compound when tested on Japanese medaka larvae for 2 days in 2, 4, 8, 16, 40, 200, and 1000 ng/L, increased liver *vtg1* gene expression and decreased *17s-hsd3*, *cyp11b*, and *11β-hsd2* gene expression in gonads [117].

Eurycomanone, found in *Eurycoma longifolia* plant extract, is a quassinoid that increases the reproductive processes of male animals. Studies report that eurycomanone increases testosterone production in rat testicular Leydig cell-rich interstitial cells by blocking aromatase and phosphodiesterase enzymes [105] and 25 mg/kg orally administered eurycomanone rich *E. longifolia* extract increases female fertility index, fecundity index, and the pup litter size [106]. Bahat and coworkers injected 0.059 and 0.118 μg eurycomanone/kg body weight and chitosan-conjugated eurycomanone to male *Clarias maggot* fish. Brain *fshβ* and *lhβ* expressions and testis *cyp11a1, star, cyp17a1, 3β-hsd, 17β-hsd, cyp19a1, ftz, ar, sox9a,* and *dmrt1* increased depending on time, dosage, or mode of application [115]. In another study conducted by the same researchers, eurycomanone and chitosan-conjugated eurycomanone was injected in female *Clarias magur* fish 3 times in 21 days, and brain *fshβ, lhβ* ve *cyp19a2* and ovary *ftz, star, cyp19a1, 3β-hsd*, *17β-hsd,* and *cyp17a1* gene expressions increased depending on dosage or mode of application [116].

Findings from all of these different studies demonstrated interactions between herbal extracts and PSMs with the regulation of different levels of the HPG axis. The changes observed in reproductive-related gene expression appear to be variable, depending on the species, mode, and duration of administration of herbal extracts and PSMs. However, herbal extracts and PSMs can influence reproduction either directly or indirectly by affecting the hormones of the HPG axis and/or by influencing apoptotic or steroidogenic pathways. The availability of sufficient steroidogenic enzymes is particularly important to support ovarian and testicular development and function, and the observed changes in gene expression of these enzymes would likely have important effects on the reproduction.

## 5. Concluding Remarks

This review summarizes some selected findings regarding the beneficial effects of herbal bioactive compounds on immune-related markers in fish species. The secondary metabolites of plants can stimulate immunity via innate and adaptive immune responses and can trigger immune cell activity, enhance phagocytosis, and enhance the secretion of inflammatory markers to resist various pathogens. Since they exhibit promising results in most investigations and have low toxicity and stressor levels, herbal bioactive compounds can be recommended as a prophylactic and therapeutic measure in the aquaculture industry. This review also reveals the direct and indirect actions of herbal extracts and phytochemicals on the reproductive axis (mainly steroidogenic pathways) in vertebrates, suggesting a potential pathway for future research.

## Figures and Tables

**Table 1:** Selected studies regarding phytochemicals and their derivatives effects on growth-related genes.

Phytochemicals/Derivatives	Dose	Type of Administration	Duration	Enhanced Gene Expression	Fish Species	Reference
Tannin	0.05 or 0.1% diet	oral	6 weeks	*gh* and *igf-i*	beluga sturgeon (*Huso huso*)	[19]
Curcumin	0.5 and 1% diet	oral	35 days	*gh*, *igf-1*, and *igf-2*	tilapia (*Oreochromis mossambicus*)	[20]
D-limonene	400 and 600 ppm diet	oral	63 days	*igf-1*, *muc*, *pept1*, *lpl* and *alp*	Nile tilapia (*O. niloticus*)	[21]
Apple cider vinegar	3 and 4.5% diet	oral	8 weeks	*GHRL*	zebrafish (*Danio rerio*)	[22]

*gh*: growth hormone; IGF-I insulin-like growth factor-I; *muc*: mucin-like protein; pept1: oligo-peptide transporter I; *lpl*: lipoprotein lipase; *alp*: alkaline phosphatase.

**Table 2:** Selected studies regarding the effects of herbal extracts and phytochemicals on immune-related markers.

Bioactive Component	Family	Dose	Type of Administration	Duration	Effect	Fish Species	Reference
*Glycyrrhiza uralensis* (*Chinese licorice*) extract	Fabaceae	6.9 mL/kg diet	Diet	60 days	Increased superoxide dismutase activity and total antioxidant capacity. Increased expression of genes *TLR2*, *TLR3*, *TLR5*, *TLR9*, *Myd88*, and *p65NFκB.* Higher expression of *IL-1β* and *IL-8* in the head kidney, not in the gill significantly. Up-regulated the expression of *IgM* and *IgD* in head kidney. Elevated disease resistance ability against *F. cloumnare* infection.	Yellow catfish (*Pelteobagrus fulvidraco*)	[53]
*Jatropha* *vernicosa* stem bark	Euphorbiaceae	50, 150 or 300 mg/L	Aqueous extract	24 h	Immunostimulant increased phagocytosis, respiratory burns activity, superoxide dismutase, and catalase activities. Increased *IL-1β* and suppressed *IL-10* gene expression potential to fight against vibriosis.	Longfin yellowtail *Seriola rivoliana* leukocytes	[54]
a mixture of garlic and lamiaceae (mint) essential oils	Amaryllidaceae and Lamiaceae	200 ppm	Diet	9 weeks	Increased fish serum lysozyme after infection. Down-regulation of *cyp11b*, *hif-1α*, *casp-3,* and *il-1β* gene expression 2 h after stress test. Up-regulated *StAR* expression.	European sea bass	[55]
leaf extract from *Salvia officinalis* (sage) and *Lippia citriodora* (lemon verbena)	Lamiaceae and Verbenaceae	MPLE; 10%, ursolic acid, 3% other triterpenic compounds; 2% verbascoside and <1% polyphenols	Extract	92 days	No significant variations of bacteriolytic and complement and IgM in cell culture, 0.1% MPLE enhanced immune response to LPS by up-regulation of *lys*, *IgM*, *tnf-α*, *il-1β*, *tgf-β1*, *il10*, *cd4*, *mn-sod,* and *cat* provides immune protection after LPS treatment.	Gilthead seabream (*Sparus aurata*) splenocytes primary cell culture	[56]
drumstick tree, *Moringa oleifera* leaf (MOL)	Moringaceae	1, 2.5 or 5%	Diet	3 weeks	Improved skin mucosal immunity including phosphatase, peroxidase, lysozyme activity, and IgM levels. Up-regulated expression of *sod*, *cat*, *tgf-β*, tight junction protein genes occludin and *zo-1*, *c3*, and *IgM* in skin and gills. MOL at the 5% level attenuated negative effects of H_2_O_2_ on the mucosal immune response in both the skin and gills.	Seabream (*Sparus aurata*)	[57]
Spirulina or selenium nano particles	Cyanobacteria	1 mg Se-NPs/kg diet, and 1 g SP/kg diet	Diet	60 days	No significant differences between fish fed Se nanoparticles or/and SP. Blood Ig M was increased by feeding both Se-NPs and SP. The transcription of liver *SOD* and *TNF-α* genes was up-regulated significantly by Se-NPs or/and SP heat shock protein 70 gene transcription was down-regulated by Se-NPs or/and SP.	Nile Tilapia (*Oreochromis niloticus*)	[58]
Sumac (*Rhus coriaria*)	Anacardiaceae	0, 0.5, 2, and 5%	Diet	56 days	Increased resistance to the pathogen WBC, RBC, lymphocyte, monocyte, and neutrophil value was significantly higher in sumac group. Serum lysozyme, and alternative complement pathway hemolytic activity and the hepatic expression of *TNF-α* and *IL-1b* were higher. mRNA expression of *IL-10* significantly decreased in fish fed 5% sumac. 2% and 5%, may effectively enhance the immune system, resistance to the pathogen, and hematopoiesis.	Rainbow Trout (*Oncorhynchus mykiss*)	[59]
Menthol essential oil after exposure with Chlorpyrifos	Lamiaceae	0.25% diet	Diet	30 days	The highest Hb, PCV, RBCs, and WBCs were observed in fish fed menthol. Had the highest total protein, albumin, and globulin, and the lowest urea, bilirubin, and creatinine after 15 and 30 days. The enzyme activities of ALP and ALT displayed low levels of menthol. Up-regulated transcription of CAT and GPX genes Menthol protected tissues from inflammation. Activated the immunity, antioxidative, and anti-inflammatory responses.	Nile tilapia	[60]
nettle (*Urtica dioica*)	Urticaceae	0.5,1 and 1.5%	Diet	8 weeks	Significantly increased expressions of *TNF-α*, *IL-1b*, *IL-6,* and *IL-8* genes. Improved growth and immunity parameters and fish resistance against *S. parasitica.*	Rainbow trout (*Oncorhynchus mykiss*)	[61]
yam (*Dioscorea oppositifolia*) extract	Dioscoreaceae	0, 0.2% and 0.4%	Diet	8 weeks	Sixteen pathways in immune system were changed significantly. Platelet activation was the most enriched pathway. Yam extract might regulate platelet activation by regulating G protein-linked receptor-mediated signal transduction and PI3K signaling pathway.	Rainbow trout (*Oncorhynchus mykiss*)	[62]
Sanguinarine from *Sanguinaria canadensis*	Papaveraceae	0,0.2,0.4, and 0.8 g/kg	Diet	8 weeks	The activity of malondialdehyde (MDA) was significantly lower. Down-regulated mRNA expression levels of *MnSOD*, *claudin*, *occludin*, *ZO-1*, and *ZO-2* significantly up-regulated mRNA expression levels of *TNF-α*, *IL-1β*, *TLR-7*, and *TLR-8.* Can enhance mRNA expression levels of genes related to intestinal immunity.	Grass carp (*Ctenopharyngodon idellus*)	[63]
*Radix bupleuri* (fennel)	Apiaceae	200, 400 and 800 μg/mL in media 0, 200, 400, 800 and 1600 mg/kg diet	Added to culture media Diet	8 weeks	Decreased serum ALP, ALT, AST, and LDH contents adipogenesis relative mRNA levels of *DGAT2*, *G6PD*, *ME1,* and *DGKα* in fish fed 200–400 mg/kg RBE diets were lower. Dietary supplementation with 200–800 mg/kg RBE diets up-regulated lipolysis-related genes (CPT1, LPL, and PPARα) expression in the liver. Dietary RBE down-regulated the expression of caspase-9, up-regulated the expression of *CAT* and *MHC2*, *IKKα,* and *TGF-β1.* Enhanced immune capability in hybrid grouper both in vitro and in vivo.	Hybrid grouper (*Epinephelus lanceolatus*♂ × *Epinephelus fuscoguttatus*♀)	[64]
Turmeric (*Curcuma longa*)	Zingiberaceae	0, 5, 10, and 15 g/kg	Diet	8 weeks	15 g/Kg^:^ lysozyme, total immunoglobulin, protein level, alkaline phosphatase, and protease activity were significantly higher. 10 g/Kg peroxidase activity was higher. Malondialdehyde level decreased significantly, expression of pro-inflammatory cytokines (*IL-1β*, *tumor necrosis factor-alpha*), signaling molecule *NF-κBp65* were down-regulated in the tested tissues in 10 and 15 g/Kg. Expression of *TLR22* was down-regulated in head-kidney and intestine in 15 g/Kg. 15 g/Kg can significantly improve the growth performance, skin mucosal and serum antioxidant parameters, and strengthen immunity.	Common carp (*Cyprinus carpio*)	[65]
*Thymus vulgaris* (thyme)essential oils	Lamiaceae	0.5, 1.0 and 2.0 mL/kg feed	Diet	2 months	1 mL: the highest up-regulation of complement component 3 and CD4 expression. 2 mL: lysozyme gene expression level significantly increased and *IL-1ß* and lysozyme genes expression were decreased. 0.5 mL: the highest survival rate was observed at 0.5 mL/kg.	Rainbow trout (*Oncorhynchus mykiss*)	[66]
*Spirulina maxima* (Microalgae)	Spirulinaceae	25 and 50 μg/mL	In embryonic water	96 h	Up-regulation of the antimicrobial enzyme (*lyz,* mucin, pro-inflammatory cytokines (*il1β*) and antioxidants (cat and sod1). The positive modulation of innate immune responses developing disease resistance against *E. piscicida* and *A. hydrophila.*	Zebrafish	[67]
*Spirulina platensis* (*Arthrospira platensis*) and Sage (*Salvia officinalis*)	Spirulinaceae and Lamiaceae	7.5 and 10 mg/kg diet	Diet	28 days	Significant increases in lysozyme, nitric oxide activities, and IgM titer with enhancement of *IL-1β* and *TNF-α* expression. Improved immune response and protected Nile tilapia against infection.	Nile Tilapia (*Oreochromis niloticus*)	[68]
Trans-cinnamic acid from *Cinnamomum verum*	Lauraceae	250, 500, 750 or 1500 mg/kg	Diet	60 days	Increased activities of phagocytic activity, respiratory burst, and potential killing. Increased the expression levels of immune-related genes SAA, *IL-8, IL-1β*, *TGF-β*, *TNF-α*, and *IgT* in head kidney of fish with 250 and 500 mg/kg. Respiratory burst activity and total antiprotease activity increased in fish fed with 500 mg/kg. Up-regulated expression of *SAA*, *IL-8, IL-1β*, *TGF-β*, *TNF-α*, *IFN-γ,* and *IgM* in fish fed 250, 500, and 750 mg/kg disease resistance against *Y. ruckeri.*	Rainbow trout (*Oncorhynchus mykiss*)	[69]
olive leaf (*Olea europea L*.) extract	Oleaceae	0.0%, 0.1%, 0.25%, 0.50% and 1.0%	Diet	60 days	*TNFα*, *IL1-β,* and *IL-8* gene expressions were significantly up-regulated. Reduced mortality. No significant changes in albumin, cholesterol, triglyceride, total protein, globulin, alkaline phosphatase, glucose, serum glutamic oxaloacetic transaminase, serum glutamate, pyruvate transaminase, and lactate dehydrogenase levels.	Rainbow trout (*Oncorhynchus mykiss*)	[70]
Drumstick (*Moringa oleífera*)	Moringaceae	0, 5, 10 and 15%	Diet	4 weeks	An improvement in head kidney leucocyte phagocytosis, respiratory burst, and peroxidase activities. Serum humoral components, including protease, ACH50 and lysozyme activities and IgM level, increased with MOL inclusion, especially at the 5% level. Improved skin-mucosal immunity such as protease, antiprotease, peroxidase, and lysozyme activities Up-regulation of the intestinal mucosal immunity genes (*lyso* and *c3*), tight junction proteins (*occludin* and *zo-1*), and anti-inflammatory cytokines (*tgf-β*) with down-regulation of pro-inflammatory cytokine (*tnf-α*).	Gilthead seabream (*Sparus aurata*)	[71]
*Rhodomyrtus tomentosa* leaf extract	Myrtaceae	1 and 10 μg/mL	Added to cell media	4 h and 24 h	Induced reduction in the expression of pro-inflammatory cytokines (*il1β, il8, and tnfα*), and increase in anti-inflammatory cytokines (*il10* and *tgfβ*), inducible enzymes (inos, *cox2*, and arginase), and an antioxidant enzyme (*gpx1*). A reduction in cellular ROS levels. Exerted immunostimulatory and anti-inflammatory effects on fish macrophages.	Rainbow trout (*Oncorhynchus mykiss*)	[72]

**Table 3:** The effect of herbal extracts and phytochemicals on reproduction-related genes.

Species/Source	Compound(s)	Species/Organ	Affected Gene(s)	References
*Genistein*	Isoflavone Angiogenesis inhibitor Phytoestrogen	Common carp *(Cyprinus carpio)* Ovary European bass (*Dicentrarchus labrax*) Scales and liver Zebrafish and Medaka (embryos)	*cyp19a1a*, liver *vtgb2*, *erβ * *vtg2*, *chgl* Zebrafish: *cyp19a1b, vtg1* Medeka: *cyp19a1a*, *vtg, cyp19a1b*	[109] [110] [111]
*Genistein and daidzein*	Genistein: Isoflavone Angiogenesis inhibitor Phytoestrogen Daidzein: naturally occurring compound in soybeans and other legumes Isoflavone	Zebrafish Ovary Testis Zebrafish Embryos-larvae Rainbow trout (*Oncorhynchus mykiss*) Juvenile	Genistein exposure: Ovary: *erβ* Ovary and testis: *HE1* Daidzein exposure: Testis: *BRDT* *esrrb, cyp1a* Liver vitellogenin, *era1, erb1*	[112] [113] [114]
*Eurycomanone* and *chitosan* conjugated *eurycomanone*	Eurycomanone: the major quassinoid in Eurycoma longifolia root extract Chitosan: a linear polysaccharide composed obtained from the outer skeleton of shellfish including lobster, crab, and shrimp	Walking catfish (*Clarias magur*) Male Walking catfish (*Clarias magur*) Female	Brain: *fshβ* and *lhβ* Testes: *cyp11a1, star,* *cyp17a1, 3β-hsd, 17β-hsd, cyp19a1, ftz, ar, sox9a, dmrt1* Brain: *fshβ, lhβ, cyp19a2* Ovary: *ftz, star, cyp19a1, 3β-hsd, 17β-hsd, cyp17a1*	[115] [116]
*Equol*	isoflavandiol estrogen metabolized from daidzein	Japanese medaka *Larvae, Liver, Gonads*	*vtg1* *17β-hsd3, cyp11b, 11β-hsd2*	[117]

## Data Availability

Data sharing is not applicable to this article.
